# Screening of the Pathogen Box for inhibitors with dual efficacy against *Giardia lamblia* and *Cryptosporidium parvum*

**DOI:** 10.1371/journal.pntd.0006673

**Published:** 2018-08-06

**Authors:** Kelly M. Hennessey, Ilse C. Rogiers, Han-Wei Shih, Matthew A. Hulverson, Ryan Choi, Molly C. McCloskey, Grant R. Whitman, Lynn K. Barrett, Ethan A. Merritt, Alexander R. Paredez, Kayode K. Ojo

**Affiliations:** 1 Department of Biology, University of Washington, Seattle, Washington, United States of America; 2 Department of Medicine, Division of Allergy and Infectious Diseases, Center for Emerging and Re-emerging Infectious Diseases (CERID), University of Washington, Seattle, Washington, United States of America; 3 Faculty of Pharmaceutical, Biomedical and Veterinary Sciences, University of Antwerp, Antwerp, Belgium; 4 Department of Biochemistry, University of Washington, Seattle, Washington, United States of America; Christian Medical College, Vellore, INDIA

## Abstract

There is need for a more efficient cell-based assay amenable to high-throughput drug screening against *Giardia lamblia*. Here, we report the development of a screening method utilizing *G*. *lamblia* engineered to express red-shifted firefly luciferase. Parasite growth and replication were quantified using D-luciferin as a substrate in a bioluminescent read-out plateform. This assay was validated for reproducibility and reliability against the Medicines for Malaria Venture (MMV) Pathogen Box compounds. For *G*. *lamblia*, forty-three compounds showed ≥ 75% inhibition of parasite growth in the initial screen (16 μM), with fifteen showing ≥ 95% inhibition. The Pathogen Box was also screened against Nanoluciferase expressing (Nluc) *C*. *parvum*, yielding 85 compounds with ≥ 75% parasite growth inhibition at 10 μM, with six showing ≥ 95% inhibition. A representative set of seven compounds with activity against both parasites were further analyzed to determine the effective concentration that causes 50% growth inhibition (EC_50_) and cytotoxicity against mammalian HepG2 cells. Four of the seven compounds were previously known to be effective in treating either *Giardia* or *Cryptosporidium*. The remaining three shared no obvious chemical similarity with any previously characterized anti-parasite diarrheal drugs and offer new medicinal chemistry opportunities for therapeutic development. These results suggest that the bioluminescent assays are suitable for large-scale screening of chemical libraries against both *C*. *parvum* and *G*. *lamblia*.

## Author summary

Development of effective single agent therapy that could be used to treat either cryptosporidiosis or giardiasis could significantly benefit those in developing regions of the world where diagnosis may be delayed or uncertain. However, asymptomatic presentations of these diseases and overlapping symptomatology with other parasitic diarrheal diseases are significant public health challenges for which there are limited available remedies. An effective drug developed based on phenotypic properties against *G*. *lamblia* and *C*. *parvum* would likely have some impact on other diarrheal parasites and be useful as a prophylactic indication against asymptomatic diseases. An important step towards this goal is the use of fast and inexpensive methods to identify new potential drug candidates. Among the goals of this study is the design of an efficient bioluminescent assay that can be used to measure growth inhibition of *G*. *lamblia* trophozoites in an efficient, high-throughput screen of large compound libraries. Stable expression of the luciferase gene introduced into *Giardia* was observed over a 24-hour incubation period. The assay was validated for reproducibility and reliability against known *G*. *lamblia* drugs with the obtained EC_50_ values within a statistically acceptable range, compared to literature values. A test of the high-throughput drug screening capability of the assay was verified in a parallel screen of *G*. *lamblia* and *C*. *parvum* with the aim of finding potential dual pathogen-inhibiting molecules. Seven compounds inhibited both parasites at low micromolar levels as confirmed by follow up assays. This series of inhibitors could be further developed as therapeutics and potentially become an important new tool for giardiasis and cryptosporidiosis treatments.

## Introduction

*Cryptosporidium* and *Giardia* are widely acknowledged as significant waterborne pathogens, as both are major contributors to the global health burden of diarrheal diseases in children under the age of five [1, 2]. Since *Giardia* and *Cryptosporidium* infections are among the most common cause of clinical and asymptomatic parasitic diseases in children in resource-limited environments, a new “dual use” therapeutic would be a very valuable treatment option. *Giardia* replicates through binary fission and colonizes the small intestine of vertebrate hosts by attachment through a ventral disk [3, 4]. This can affect the host’s ability to effectively absorb necessary nutrients [5]. Young children can suffer physical stunting and wasting, cognitive impairment, and fine motor movement problems [5, 6]. Food and Drug Administration (FDA) approved treatments for giardiasis include metronidazole, chemically related nitroimidazole drugs, and albendazole. However, a substantial number of clinical presentations are resistant to these treatments [7, 8]. A combined treatment regimen of metronidazole and albendazole or quinacrine can be highly effective for patients with metronidazole-resistant giardiasis [9, 10] but toxicity often limits therapy. *Cryptosporidium* infections are chronic and, in some cases, fatal in immune compromised patients [11–14]. Infection is caused by ingestion of environmentally resistant infective stages, called cysts (*G*. *lamblia*) or oocysts (*C*. *parvum*), upon passage in the feces [15]. Ingestion of as few as 10 cysts or oocysts can lead to clinical episodes [16–18]. Nitazoxanide is the only FDA-approved therapy for cryptosporidiosis. However, nitazoxanide has shown little efficacy in immunocompromised patients [19] and is not approved for use in children under the age of one [20]. Consequently, there is an increasing need to develop alternative drugs for both cryptosporidiosis and giardiasis [21].

Methods to accelerate drug discovery by high-throughput screening of large chemical libraries will require development of assay platforms that provide reliability, sensitivity, have easily observable readouts, and are both cost and time efficient. A bioluminescent assay that fits these criteria for high-throughput drug screening of inhibitors against *C*. *parvum* was recently described [22, 23]. Screening of molecules against *G*. *lamblia* parasites traditionally involved microscopic counting of parasites [24], or utilizing a MOXI cell coulter counter [25], both of which require manual counting of each well in an assay plate. Semi high-throughput assays using resazurin to measure cell viability [26] and another based on automated image analysis by cell stained-DAPI signal read-out have also been explored [27]. Most recently, a digital phase-contrast microscopy morphology-based assay method with enumeration by software was developed, which does not require cell staining [28]. The morphology-based assay is comparable to the previously reported DAPI stain method, but it relies solely on expensive software to identify and count parasites based on size and morphology [27, 28]. Stable expression of *Escherichia coli* β-glucuronidase A (GusA) as a reporter gene for *G*. *lamblia* growth measurement was also described as suitable for high-throughput drug screening [29]. We now describe the development of an efficient bioluminescent assay for measuring growth inhibition of trophozoites using an engineered *G*. *lamblia* strain expressing a red-shifted firefly luciferase PpyRE9h gene [30].

The Pathogen Box (www.pathogenbox.org; Medicines for Malaria Venture (MMV), Geneva, Switzerland) is a set of 400 molecules directed to a variety of “neglected disease” pathogens, including *Plasmodium*, *Mycobacterium tuberculosis*, helminths, kinetoplastids, *Cryptosporidium*, *Toxoplasma*, and dengue virus. Among the 400 molecules are 26 reference compounds [31] that are known bioactives against particular pathogens (https://www.pathogenbox.org/about-pathogen-box/composition). The Pathogen Box was screened for activity against *G*. *lamblia* and *C*. *parvum* cells, in an attempt to find potential dual pathogen-inhibiting molecules.

## Materials and methods

### Chemical inhibitors

The Pathogen Box (MMV) molecules were obtained as 10 mM DMSO stocks and stored at -20 °C. Metronidazole (Sigma, St. Louis, MO), a commercially available drug, was included in the study as a preliminary control of the assay, while quinacrine was used in cytotoxicity screening.

### Parasite cultures

*Giardia lamblia* (WBC6, ATCC 50803) trophozoites were grown in TYI-S-33 medium supplemented with 10% bovine serum and 0.05 mg/mL bovine bile [32]. Transgenic *C*. *parvum* strain UGA1 expressing Nanoluciferase (Nluc) [22] used in this study was grown in HCT-8 cells. Cultures were incubated at 37°C.

### *G*. *lamblia* expression construct, electroporation and selection of luciferase expressing parasite strains

Oligonucleotide pairs used to generate the *Giardia* transfection construct are the RE9 primers (5' ACCATGGAATCTAGAATGGAGGACGCCAAGAACAT 3' and 5' TGGATCCTCTTAATTAATCAGATCTTGCCGCCCTTCTTGGCC 3'); pBetaTub-Gib-forward (5' TCTGCAAGTTAATTTTTGGCCCCTAGGTCGGATCAAGACTTCAAATTAGAAA 3') and BetaTubUTR-Gib-reverse (5' TTATTTGACCATCGTACTTGCAACTAGTGAGCTCGGTACCAGCTGATCGGCGC 3'). The expression plasmid (pBetaTubulinPro::PpyRE9h::BetaTubulin3'UTR/pPAC-integ) was constructed by cloning the PpyRE9h luciferase gene downstream of a *β*-Tubulin promoter. First, the entire open reading frame of the RE9hum (thermostable red-shifted firefly luciferase *PpyRE9h*) was digested from the parent plasmid vector pTRIX2-RE9h using XbaI and BamHI. The RE9hum fragment, representing the 2.8 kb region, was amplified and subcloned into mNeonGreen-C18-β-tubulin [33] after excising the tubulin gene and mNeonGreen with NcoI and BamHI and digesting the amplicon with the same enzymes forming pβTub::PpyRE9h::βTub3’UTR. The pβTub::PpyRE9h::βTub3’UTR fragment was amplified using Phusion High-fidelity DNA polymerase (Thermo Fisher Scientific, Rockford, IL) and cloned into pPacV-integ digested with EcoRI and PmlI, using Gibson cloning. The vector pβTub::PpyRE9h::βTub3’UTR/pPacV-integ was used to integrate into chromosome 5 by homologous recombination [34]. For integration, 30 μg of pβTub::PpyRE9h::βTub3’UTR/pPacV-integ was linearized with SwaI overnight, precipitated with ethanol and incubated with 300 μL of chilled *G*. *lamblia* cells (~13x10^6^/mL) for 30 minutes before electroporation (Bio-Rad GenePulser X at 375 V, 1000 uF, 750 ohms). After electroporation, cells were incubated on ice for 10 minutes before being transferred to fresh media at 37 °C. Transfectants were selected with puromycin after overnight recovery, as previously described [35].

### Assay parameters determination for *G*. *lamblia*

To determine optimal D-luciferin (Gold BioTechnology Inc., St. Louis, MO) concentration, substrate was serially diluted 1:2 in PBS from 100 mg/mL to 0.052 mg/mL. Parasite numbers were kept constant at 50,000 cells/mL in a volume of 250 μL per well. Plates were incubated for 30 minutes at 37 °C on a plate shaker. Wild-type *G*. *lamblia* (non-luciferase expressing) was used as a control.

To determine the optimal parasite concentration, an initial cell concentration of ~1x10^6^ cells/mL was serially diluted 1:2, to a minimum concentration of ~500 cells/mL. Luminescence was measured at 20, 30 and 40 minutes in white polystyrene, flat bottom 96-well plates (Corning Incorporated, Kennebunk, ME) on an EnVision plate reader (PerkinElmer, Waltham, MA).

### *G*. *lamblia* luciferase strain assay condition validation with metronidazole

Preliminary effective concentration that causes 50% growth inhibition (EC_50_) assays were performed on the luciferase expressing and wild-type (control) *G*. *lamblia* cells to establish a correlation between manually counted cells and the bioluminescent signal read out. Trophozoites were harvested by chilling cultures on ice for 30 minutes and plated by adding 150 μL per well at a concentration of 250,000 cells/mL. Metronidazole (Sigma, St. Louis, MO) was used as a control and was serially diluted 1:2 from 40 μM to 0.04 μM and 150 μL was added to the 150 μL of the *G*. *lamblia* trophozoites. Plates were covered in plastic low-evaporation lids, sealed in individual anaerobic BD GasPak Bio-Bags (Becton Dickinson, San Jose, CA) and incubated at 37 °C for 24 hours. Plates were then iced for 30 minutes and 250 μL of resuspended cells were transferred to a solid white 96-well plate and 50 μL of 10 mg/mL D-luciferin was added to a final reaction concentration of 1.67 mg/mL. Plates were incubated on a plate shaker at 37 °C for 30 minutes and read on an EnVision Plate Reader. A duplicate plate was concurrently set up and assayed using a MOXI Z Mini Automated Cell Counter Kit (Orflo, Ketchum, ID). Results obtained from the bioluminescence assay and the MOXI counter data were compared to evaluate their correlation to each another. Albendazole (Sigma, St. Louis, MO), an alternative giardiasis therapeutic, was assayed in concentrations from 4 μM to 0.004 μM with only the luciferase expressing *G*. *lamblia* cells.

### *In vitro* activity of compounds against *C*. *parvum*

Growth inhibition and EC_50_ determination assays were performed using UGA1 Nluc expressing *C*. *parvum* strain in HCT-8 cells (ATCC^©^, Manassas, VA) as previously described [23]. HCT-8 cells were inoculated onto 96-well plates and allowed to grow for 48 hours to 90–100% confluence and then treated with single concentration (10 μM) or serially diluted compounds prior to oocyst infection. *C*. *parvum* oocysts were incubated for 10 minutes in 10% bleach at room temperature and then washed with DPBS. One thousand oocysts per well were applied to the 96 well plates with RPMI-1640 medium supplemented with 10% horse serum and 1% penicillin/streptomycin at the same time as compound addition and incubated for 48 hours. The cell monolayers were lysed for 1 hour before adding Nano-Glo^®^ luciferase reagent (Promega, Madison, WI), and measuring relative luminescence units (RLU) in an EnVision Plate Reader (Perkin Elmer, Waltham, MA). Several parameters, including reproducibility and reliability, were previously evaluated to optimize this assay for low and high-throughput (HTP) screening of small molecule compounds against *C*. *parvum* [23]. EC_50_ values were calculated using Prism 7 software (GraphPad Software, San Diego, CA).

### Pathogen Box molecule screening

The 400 compounds in the Pathogen Box were initially screened against the bioluminescent strain of *G*. *lamblia* in duplicate at 16 μM as described above. Further experiments were carried out to determine the EC_50_ values of molecules that were found to have ≥ 95% inhibition of *G*. *lamblia* or *C*. *parvum* cell growth after the initial screen. Compounds were serially diluted 1:2 starting at an initial concentration of 8 μM to a final 0.015 μM for *G*. *lamblia*. *C*. *parvum* screening against the Pathogen Box compounds was performed as previously described starting at a concentration of 10 μM [23]. Assays to determine EC_50_ values were also performed for a subset of compounds found to have ≥ 75% growth inhibition of both *G*. *lamblia* and *C*. *parvum* in the initial screens as potential dual inhibiting molecules.

### Cytotoxicity against HepG2 cells

Mammalian cell cytotoxicity assay data performed on HepG2 (human hepatocellular carcinoma) cells and described as CC_20_ or CC_50_ values are provided in supporting documentation that accompanied the Pathogen Box for approximately three quarters of the 400 compounds. However, no details of the assay conditions or parameters were provided [31]. In this study, selected compounds were further tested for cytotoxicity against HepG2 cells as previously described [36]. Briefly, cells were exposed to two-fold serial dilutions of compounds for 48 hours and cytotoxicity was assessed using the AlamarBlue^®^ viability assay (Thermo Fisher Scientific, Waltham, MA). Each dilution was assayed in quadruplicate and concentrations causing 50% growth inhibition (CC_50_) were calculated by non-linear regression using GraphPad Prism 7 software (GraphPad Software, San Diego, CA).

### Statistical analysis

The data was analysed in Microsoft Excel and Prism 7 software (GraphPad Software, San Diego, CA). A nonlinear regression sigmoidal dose-response curve fit was applied to dose-response data for both half maximal effective concentration and half maximal cytotoxic concentrations.

## Results

### Red-shifted luciferase expressing *G*. *lamblia* strains and assay parameter optimization

**Fig 1:**
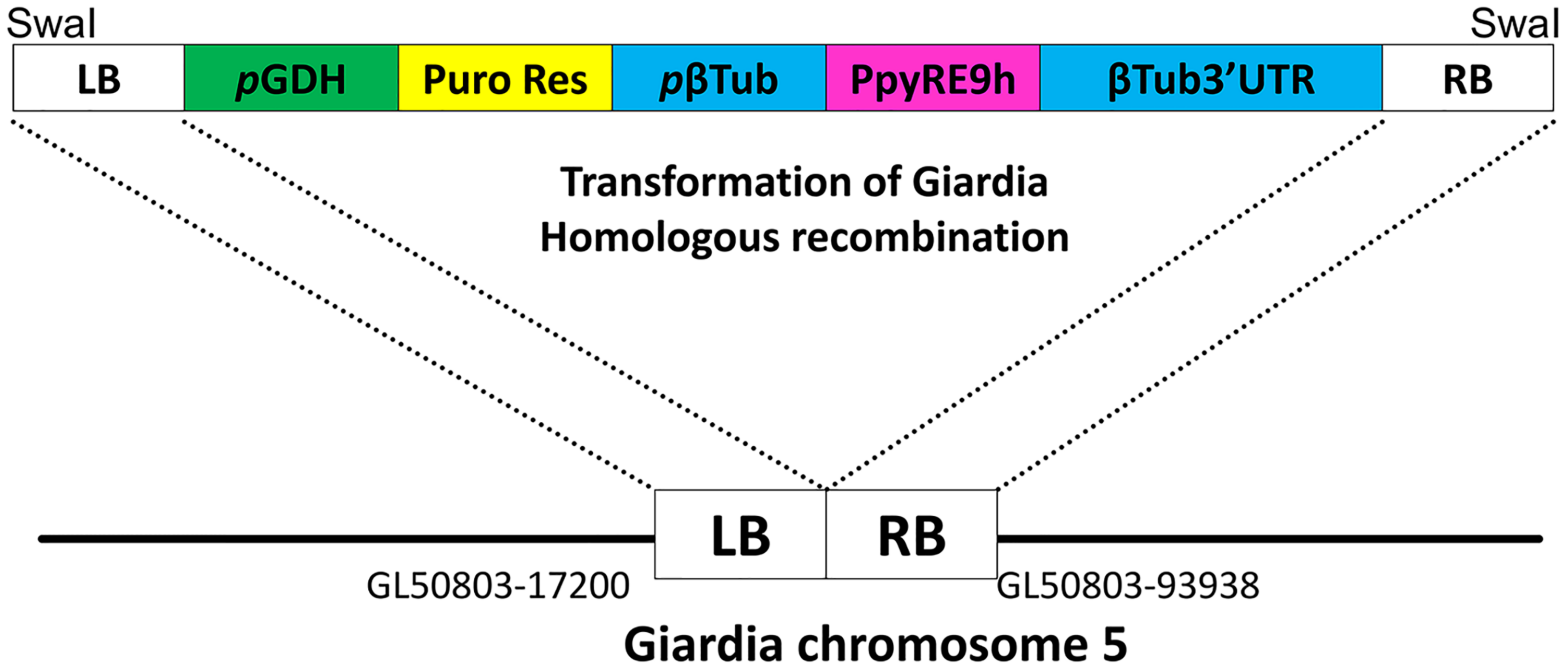
Schematic of targeting vector and homologous recombination. The expression cassette containing pGDH::Puro Res and pβTub::PpyRE9h:: β3’TubUTR was used for homologous recombination to target a genomic intergenic region which is flanked with GL50803_17200 and GL50803_93938. After electroporation, puromycin was used to select for transfected cells. LB = left border, pGDH = Promoter of glutamate dehydrogenase (GL50803_21942), Puro Res = puromycin N-acetyltransferase, pβTub = β tubulin promoter, PpyRE9h = red-shifted luciferase coding sequence, β3’Tub UTR = β tubulin 3’UTR, RB = right border.

**Fig 2:**
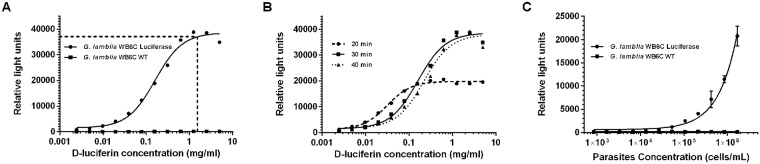
Assay parameters determination. **A**. D-luciferin concentration in relation to RLU. Serial concentration of D-luciferin relative to the wild-type strain to determine the optimal concentration of D-luciferin to use in assays. The reaction was carried out for 30 min. The line shows the RLU data-points at 10 mg/ml of D-luciferin. (RLU: relative light units). **B**. Optimization of incubation time. D-luciferin concentration in relation to relative light units (RLU) was measured at different time points. **C**. Parasite concentration and RLU values. The plot showed bioluminescence read out from the wild type strain and the transfectant strain correlating number of cells to RLU. The values of both the luciferase strain and wild-type strain are shown with respective RLU at 30 min. (P-value of luciferase strain < 0.001: Wild-type p = 0.854). RLU versus parasite concentration helps to determine the ideal maximal end concentration without overgrowth carrying out the reaction for 30 min.

Wild-type *G*. *lamblia* cells were transfected with pβTub::PpyRE9h::βTub3’UTR/pPacV-integ (Fig 1), selected clones express red-shifted firefly luciferase *PpyRE9h* as validated by detectable bioluminescence signals when incubated with serial concentrations of D-luciferin in parallel to the wild-type strain (Fig 2a). Optimum luminescence was achieved after 30 minutes of incubation with D-luciferin (Fig 2b). The optimal concentration of D-luciferin was determined to be 1.67 mg/mL, as bioluminescence signals plateaued above this concentration within 30 minutes (Fig 2b) demonstrating no reasonable need for higher concentrations. The amount of red-shifted firefly luciferase-driven bioluminescence activity was proportional to the number of viable transfected *G*. *lamblia* parasites per well, as determined by a comparative analysis with direct cell count of both the wild type and transfectant strains (Fig 2c). Considering *G*. *lamblia’s* doubling time of 8 hours [33], the optimal starting concentration of cells to achieve the highest bioluminescent signal was determined to be ~125,000 cells/mL, as this would result in an ending concentration of ≥10^6^ cells/mL after 24 hours of growth (Fig 2c).

### Assay condition validation with metronidazole and albendazole

**Fig 3:**
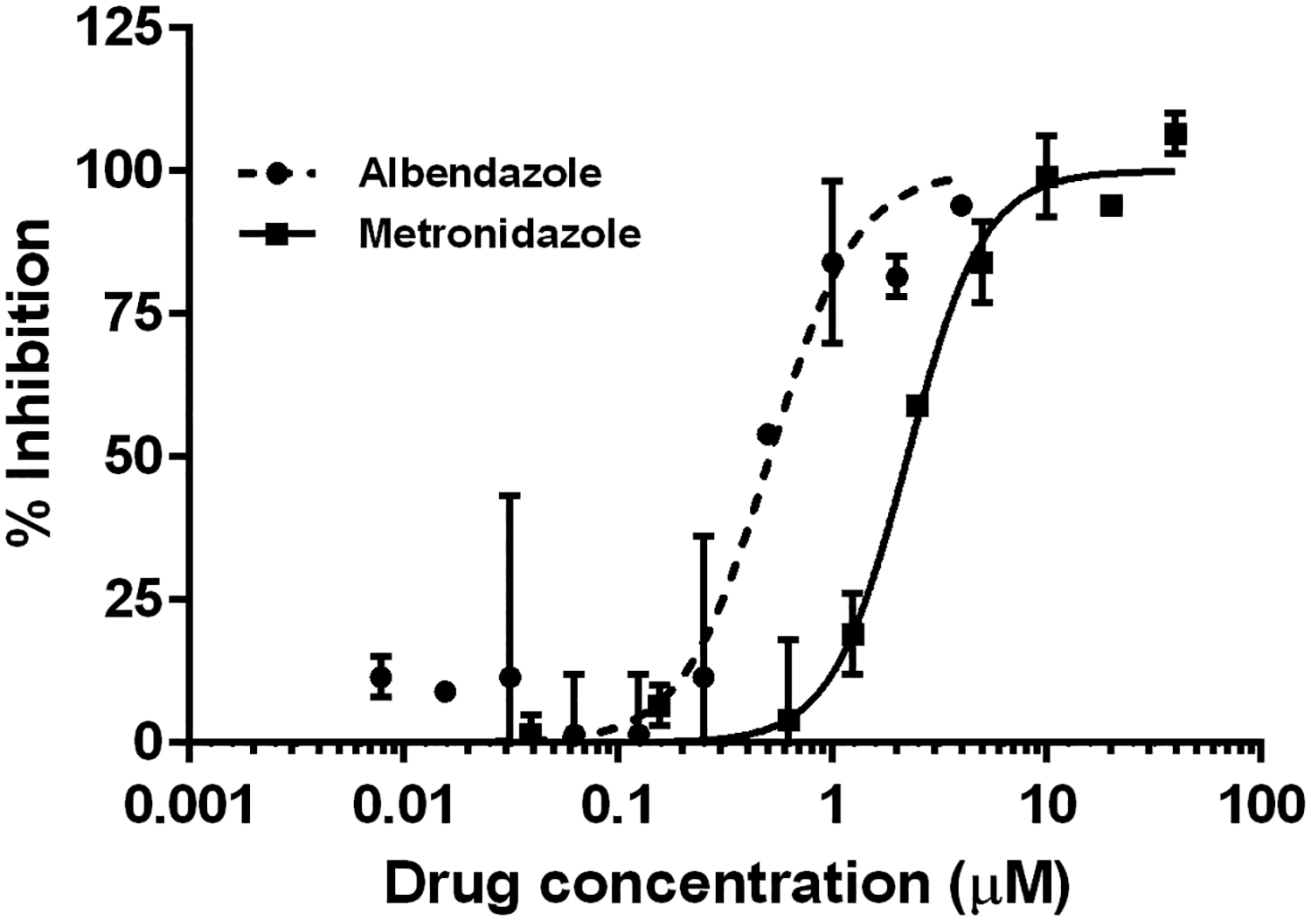
A plot of the EC_50_ of values of metronidazole and albendazole. Growth of red-shifted luciferase expressing *Giardia* trophozoite was inhibited by metronidazole and albendazole with EC_50_ of 2.24 μM and 0.41 μM, respectively.

A preliminary experiment to determine the reliability of the bioluminescence-based *G*. *lamblia* assay for drug inhibitory activities was performed using metronidazole and albendazole. The metronidazole EC_50_ values obtained from 125,000 cells/mL by MOXI coulter counter (EC_50_ = 1.06 μM) and the luciferase-based assay (EC_50_ = 2.24 μM) are within the acceptable range for experimental errors. The luciferase-based assay EC_50_ value of 0.41 μM was obtained for albendazole (Fig 3). These EC_50_ values confirmed the reliability of the luminescence signal-based assay as compared to previous cell counting methods. Furthermore, the experimentally derived EC_50_ value of standard clinical giardiasis therapies using this new assay track with previously documented literature values of ~5 μM [37] or 8.65 μM [38] for metronidazole and 0.23 μM for albendazole (reference strain—WB) [38].

### Initial screening hits and EC_50_ against *Giardia and Cryptosporidium*

The screening of the Pathogen Box against *G*. *lamblia* at 16 μM led to the identification of one hundred and four compounds with ≥ 50% inhibition, forty-three compounds with ≥ 75% inhibition of cell growth, among which twenty-two showed ≥ 90% and fifteen compounds showed ≥ 95% inhibition (S1 Table and Table 1). EC_50_ values determined for thirteen of the molecules with ≥ 95% inhibition ranged from 0.5 to >10 μM (Table 1). Two (MMV153413 and MMV688283) of the fifteen hits with ≥ 95% inhibition at 16 μM initial screening were deemed to be false positives since they were not potent in the follow up serial titration of the drugs. Screening against *C*. *parvum* at 10 μM yielded one hundred and eighty-five compounds with >50% inhibition, eighty-six compounds with ≥75% inhibition, twenty-five compounds with ≥ 90% inhibition and six compounds with ≥ 95% inhibition (S1 Table). The six compounds with cell growth inhibition ≥95% were tested to determine their EC_50_ values against *C*. *parvum*. The compounds MMV676477, MMV688853, MMV002817, MMV085499, MMV019189 and MMV102872 have *C*. *parvum* EC_50_ values of 0.22, >10, 0.48, 1.93, 2.52, and 0.14 μM respectively.

**Table 1:** Pathogen Box compounds tested for dose response against *G*. *lamblia*.

Common name	MMV Compound ID	*G*. *lamblia* EC_50_	HepG2 CC_50_ (μM) (this study)	HepG2 CC_50_ (μM) (literature values)
		(μM)	(μM)	
Nifurtimox	MMV001499	0.64 ± 0.12	>40	>80*^
N/A	MMV022478	2.41 ± 0.12	>40	>10*
N/A	MMV028694	3.88 ± 1.27	15.87	8.10*
N/A	MMV495543	2.77 ± 0.21	>40	47.60*
N/A	MMV676395	1.57 ± 0.20	>40	>100*
Clofazimine	MMV687800	1.79 ± 0.23	26.43	>20^+^
N/A	MMV687807	0.51 ± 0.06	<5	0.70*
N/A	MMV687812	1.25 ± 0.12	5.33	3.90*
Delamanid	MMV688262	0.55 ± 0.04	>40	72.50*
N/A	MMV688755	1.57 ± 0.38	>40	>50*
N/A	MMV688844	2.30 ± 0.43	>40	>50*
Auranofin	MMV688978	3.74± 0.46	0.50	4.50^#^
Nitazoxanide	MMV688991	0.80 ± 0.10	10.73	6.35*^

* Pathogen_Box_Activity_Biological_Data_ Structure & Smiles (https://www.pathogenbox.org/about-pathogen-box/composition)

N/A–not applicable;

^ HepG2 CC_20_ (μM)

^+^ reference [41];

^#^ reference [56]

Of the 400 Pathogen Box molecules, there were sixteen molecules with activity against both parasites at ≥75% inhibition. EC_50_ analysis was performed for a representative subset of seven of these dual hit molecules. All seven molecules had EC_50_ values of ≤ 5 μM on both parasites within the selected cytotoxicity criteria (HepG2 ≥5-fold of the observed EC_50_ value). Previously described anti-giardia and/or anti-cryptosporidiosis drugs auranofin [39, 40], nitazoxanide [41], nifurtimox [38], and clofazimine [41] were found as dual hits in this screen. Furthermore, EC_50_s obtained using the new assay platform are very similar to those obtained for the wild-type *G*. *lamblia* strain and available literature-based comparisons (Table 2, S2 Table). Auranofin is highlighted as a potent hit against *G*. *lamblia* and *C*. *parvum* (Table 2), in agreement with literature results (*G*. *lamblia* EC_50_ = 4–6 μM in [40]; *C*. *parvum* EC_50_ = 2 μM in [39]). Clofazimine (*G*. *lamblia* EC_50_ = 1.79 μM and *C*. *parvum* EC_50_ = 3.3 μM) is a promising dual treatment option to further explore. The remaining tested hits (Table 2) share no obvious chemistry with these previously characterized anti-parasitic drugs. One of the best examples of this novel dual-inhibiting compound is MMV010576 which had 3.0 and 1.9 μM EC_50_ against *C*. *parvum* and *G*. *lamblia* in cell proliferation assays, respectively (Table 2). The other is MMV676501 with detectable cytostatic effects on mammalian cells at high concentrations of 50.1 μM (Table 2). These have reasonable safety indices (EC_50_ of both parasites/CC_50_ against mammalian cells) for further exploration.

**Table 2:** Representative set of dual hits against *Giardia* and *Cryptosporidium* with ≥ 75% inhibition. EC_50_: 50% effective concentration, CC_50_: 50% cytotoxic concentration, % inh: percentage inhibition.

Common name	MMV Compound ID	*G*. *lamblia* EC_50_ (μM)	*C*. *parvum* EC_50_ (μM)	HepG2 CC_50_ (μM) (this study)	HepG2 CC_50_ (μM) (literature values)
Iodoquinol	MMV002817	2.52 ± 0.45	0.48± 0.2	~40	2.50*^
N/A	MMV010576	1.9 ± 0.28	2.99 ± 2.1	>40	>10*
N/A	MMV028694	3.88 ± 1.27	1.56 ± 0.5	15.87	8.10*
N/A	MMV676501	1.40 ± 0.2	4.96 ± 1.8	>40	50.10*
Clofazimine	MMV687800	1.79 ± 0.2	3.33 ± 1.2	26.43	>20^+^
Auranofin	MMV688978	3.74 ± 0.46	3.34 ± 2.7	0.50	4.50^#^
Nitazoxanide	MMV688991	0.80 ± 0.10	3.10 ± 0.4	10.73	6.35*^

* Pathogen_Box_Activity_Biological_Data__ Structure & Smiles (https://www.pathogenbox.org/about-pathogen-box/composition)

N/A–not applicable;

^ HepG2 CC_20_ (μM)

^+^ reference [41];

^#^ reference [56]

There were no specific data on the biological activity of inhibitors against *G*. *lamblia* as documented for *C*. *parvum* in the MMV Pathogen_Box_Activity_Biological_Data_Smiles (https://www.pathogenbox.org/about-pathogen-box/composition). This makes direct comparison of biological activity for *G*. *lamblia* obtained in our study against the Pathogen Box data sheet challenging. However, 11 compounds in the Pathogen Box series were designated as anti-cryptosporidiosis agents. Pathogen Box datasheet reported EC_50_s between 0.05 μM and >5 μM for compounds MMV675968, MMV675993, MMV675994, MMV676050, MMV676053, MMV676599, MMV688853 and MMV688854. Initial screening data in our study found *C*. *parvum* cell growth inhibition at 10 μM to be between 79 and 96% with substantially lower effects on *G*. *lamblia* trophozoites (S1 Table). Hence, they were not progressed to the next stage of EC_50_ determination based on our dual hit selection criteria. Compound MMV675968 was however, a dual hit in the initial screen with 88 and 90% inhibition of *C*. *parvum* and *G*. *lamblia* growth, respectively. In the follow up serial dilution assays, MMV675968 had EC_50_s of 2.1 μM and >10 μM for *C*. *parvum* and *G*. *lamblia*, respectively, thus ruling it out as a dual hit. MMV676191 had a 36% inhibitory activity on *C*. *parvum* cells in our initial screen at 10 μM, in agreement with the Pathogen Box datasheet reported EC_50_ of >10 μM. Dual hit compounds MMV676604 (*C*. *parvum* EC_50_ of 2.9 μM) and MMV676602 (*C*. *parvum* EC_50_ of 0.31 μM) were also not effective in the follow up EC_50_ assay against *G*. *lamblia*. The most obvious conclusion from the comparative analysis of anti-Cryptosporidiosis activity as provided by the MMV Pathogen Box datasheet with those obtained in this study is that there is great overlap in the results against *C*. *parvum* cells.

### Cytotoxicity

Compounds with parasite EC_50_s ≤5 μM and within the selected cytotoxicity criteria (on mammalian HepG2 cells) were deemed potent hits. Sixteen compounds are dual hits with ≥75% initial growth inhibition against both parasites, three molecules were dropped from further consideration due to HepG2 cytotoxicity data from MMV and reduced efficacy in the follow up assay to determine their EC_50_s for *G*. *lamblia*. These include compounds MMV676602 and MMV676604. Recent interest in compound MMV687807 as a potential compound that may be further developed as an agent against *Candida albicans* [42] and *Toxoplasma gondii* [43] despite undesirable activity at 0.7 μM against HepG2 (Table 2) argues for its inclusion in our analysis against *G*. *lamblia*. However, MMV687807 was too toxic to the HCT-8 host cells in the *C*. *parvum* assay that a reliable EC_50_ value could not be determined. Although auranofin showed high cytotoxicity levels of 0.50 μM against HepG2 cells (Tables 1 and 2), it has already entered clinical trials as a possible anti-giardial [44]. Similarly, nitazoxanide showed activity against *C*. *parvum* and was considered a legitimate hit being already approved for clinical use. Altogether, seven molecules with EC_50_s ≤5 μM and within the selected cytotoxicity barrier (in HepG2 ≥5-fold of the observed EC_50_) were deemed potent dual hits.

## Discussion

Despite the significant health impact of giardiasis and cryptosporidiosis, research into new drug treatments are only beginning to move ahead with promising strategies and new drug leads [45]. Cryptosporidiosis and giardiasis illness can be self-limiting or persistent without overt symptoms, but still exert a detrimental effect on growth [46]. When these infections become symptomatic, the overlap in symptomatology with a host of other enteric infections is plentiful. Co-infection of *Cryptosporidium* and *Giardia*, as well as co-infections with other pathogens, have been reported in studies from a number of developing countries [46–48] where rapid diagnostics may be available but not widely used. Even in resource-rich communities where specific diagnostics could be used routinely, definite identification of etiologic agent of mixed parasitic infections each with overlapping symptomatology could be challenging. The potential for devastating consequences of giardiasis and cryptosporidiosis in immunocompromised patients and malnourished children as clinical or asymptomatic presentations emphasize the need for an effective therapy that could be used as point of care treatment. This point of care treatment will be appropriate where diagnosis may be delayed or uncertain. Such treatments could be useful as a prophylactic in combating asymptomatic presentations in regions with lagging sanitary conditions. An important step towards this goal is the use of fast and inexpensive methods to identify new potential drug candidates. A recently described bioluminescence assay [22, 23] effectively solved this problem for *C*. *parvum*. The luciferase expressing strain of *G*. *lamblia* described here offers a similar tool for use in the search for new giardiasis treatments.

The assay described here may be compared to a previously reported ATP-based screening in *G*. *lamblia* [38] that employed ATP lite, a Luminescence ATP Detection System (Perkin Elmer) that requires a cell lysis step and exogenous supplementation of the reaction mixture with luciferase protein and D-luciferin in a well-controlled pH environment, to ensure no endogenous ATP degrading enzyme activity [38]. The engineered luciferase expressing *G*. *lamblia* strain described here employs the same principle. However, it has the advantage of requiring neither a lysis step nor exogenous luciferase supplementation since this *G*. *lamblia* strain constitutively expresses the luciferase needed for the read out, thereby reducing the resources needed for screening.

We have shown here that the engineered *Giardia* strain PpyRE9h-Tub is an effective tool for phenotypic screening of compounds with potential anti-giardial activity. The bioluminescence measurement provided by this strain offers similar efficiency when adapted to high-throughput screening as the previously described GusA *G*. *lamblia* [29]. Strain PpyRE9h-Tub has additional benefit for a broader range of assays, in particular the potential for non-invasive imaging of parasite load in *Giardia*-infected animals. Evidence of use of the luciferase reporter in a mouse model of giardiasis was recently reported [49]. Comparative analysis showed that the luciferase-based assay described here has a high level of reliability and reproducibility based on the assay of a known standard drug, metronidazole, a previously-known alternative, albendazole, and determination of EC_50_s for selected hits from the Pathogen Box library. This assay overcomes the limitation of drug screening platforms that rely on assessment of parasite numbers without regard to parasite viability [27, 28], since only viable parasite cells can produce the ATP required for the D-luciferin reaction.

One limitation of the luminescence screening platform is less efficient parasite growth in volumes lower than 300 μL per well in 96-well plates, due to *G*. *lamblia*’s surface area to volume requirement. Lower reaction volume seems to result in higher oxygen exposure during handling that prevents optimum cell replication [27]. The luciferase assay is further constrained by only detecting the bioluminescent signal, while the digitized methods, using DAPI or phenotypic recognition, both have potential for retrospective analysis of cytology [28]. However, this could be more appropriately re-evaluated further downstream during drug development. The set-up of the assay as described here includes a relatively high inoculum incubated for 24 hours. This may have the disadvantage of missing compounds with slower mode of action, which may explain why reference compounds with known efficacies against *G*. *lamblia* such as mebendazole (MMV003152) and pentamidine (MMV000062) did not show up in the screen with the luciferase readout described here. This can be remedied with a set-up using lower inoculum for a longer incubation time (48 hours). An unfortunate shortcoming of cell based high-throughput screens is the poor correlation that is sometimes observed between percent inhibition at fixed concentration (initial screen) and the EC_50_ obtained in a dose response assay. Hence, the initial dual hit MMV024114 as well as a number of hits were not potent in the follow up serial dilution assays to determine their EC_50_s for *G*. *lamblia* (MMV675968, MMV676602, MMV676604 and MMV688283) and *C*. *parvum* (MMV024406 and MMV676395). Previous studies have shown that false positive and/or negative rates could be high [50]. Furthermore, assigning cut off points for the selection of inhibitors for further evaluation may sometimes miss compounds that perform excellently in vivo. An example in this study is the calcium dependent protein kinase inhibitor, BKI-1294 (MMV688854), with 79% inhibition at the initial screening concentration of 10 μM which is not within the 95% cut off. BKI-1294 (MMV688854) was previously shown to be effective (EC_50_ 2.7 μM) using the same strain of Nluc *C*. *parvum* in an in vivo mouse model of cryptosporidiosis [23]. Nonetheless, the bioluminescent assay is an efficient and inexpensive method for high-throughput screening that does not require counting, staining, or lysis and can be further used to study efficacy with in vivo models of infection [49, 51].

The top hits from screening against *G*. *lamblia* trophozoites (Table 1) include two drugs, nitazoxanide and nifurtimox, which share the same free-radical mediated mode of action as the current front-line anti-giardial metronidazole. Both have been previously noted for potential anti-giardial use [41, 52] and nitazoxanide has been used against *C*. *parvum* demonstrating some clinical efficacy including a significantly lower mortality rate in one trial [53]. There is a strong potential for cross resistance since these compounds share their mechanism of action with metronidazole [41, 45].

Another known drug identified in the screen, iodoquinol, is primarily used for anti-amoebiasis but is also used as an anti-giardial drug, in combination with metronidazole [54]. Iodoquinol chelates ferrous ions that are essential for metabolism in amoeba. To the best of our knowledge, it had not been used for treatment of cryptosporidiosis, but can be included as a candidate for dual use.

The FDA-approved drug auranofin has gained interest in regards to a possible repurposing strategy due to its anti-parasitic activities in *S*. *mansoni*, *T*. *brucei*, *E*. *histolytica* and *G*. *lamblia* [40]. Auranofin was previously identified as potent against *C*. *parvum* [39] in screens to re-profile FDA-approved drugs for new therapy for neglected tropical diseases. The mechanism of action of auranofin mainly consists of inhibiting reduction/oxidation enzymes, thereby damaging pathogens by oxidative stress. Auranofin activity against *C*. *parvum* and *G*. *lamblia* trophozoites as shown here (Table 2) is consistent with previous findings of its usefulness as a broad anti-diarrheal agent [39]. It has already entered clinical trials as a possible anti-giardial [44]. Auranofin and iodoquinol provide the best argument for the possibility of drug candidates with therapeutic effects on multiple diarrheal parasites that can be carefully deployed for syndromic use at the point of care.

Clofazimine also shows promise for use in treatment of both cryptosporidiosis and giardiasis. Good clinical outcomes in experimental models have increased enthusiasm for clofazimine as a potential drug repurposing candidate for the treatment of human cryptosporidiosis [41]. It is currently being evaluated in clinical trials as a potential anti-cryptosporidiosis agent. It would be of scientific interest to further study clofazimine’s efficacy and safety for treatment of both pathogens, as it is a regulatory approved drug that has been used for over 50 years for leprosy and more recently for the therapy of multi-drug resistant tuberculosis.

MMV010576 selectively kills both parasites with little to no cytotoxicity to mammalian cells. The scaffold for this compound was originally identified from a high throughput screen of a BioFocus DPI SoftFocus kinase library of selective anti-plasmodial hits. MMV010576 belongs to a novel class of orally active 3,5-diarylaminopyridine series, which combines good in vitro activity against *P*. *falciparum* with efficacy in a *P*. *berghei* mouse model following administration of single oral doses [55]. MMV010576 is a kinase inhibitor [31] found to have selective inhibition of *C*. *parvum* and *G*. *lamblia* cell proliferation relative to a mammalian cell line (Table 2). The properties of MMV010576 provide preliminary evidence that dual parasite inhibiting kinase inhibitors could be identified in a phenotypic HTS assay. It raises the potential for development and use of kinase inhibitors for syndromic treatment of either cryptosporidiosis or giardiasis. Together with three similarly uncharacterized hits retrieved from recent screening of the Malaria Box [28], these constitute a valuable starting point for the development of dual anti-giardial and anti-cryptosporidiosis drugs.

### Concluding remarks

The bioluminescent assay for *Giardia* growth described here was optimized and found to be comparable to assays performed with automated cell counters. Reproducibility was validated by comparing known literature EC_50_s of metronidazole, auranofin, nitazoxanide and nifurtimox, finding all to be within a two-fold difference of our results. This suggests that the described bioluminescent assay can efficiently be used for semi high-throughput drug screening. Furthermore, the found dual-inhibiting compounds are of great public health importance since these could be suitable for co-infections or in situations where the precise diarrheal-causing pathogens cannot be determined. The luciferase-expressing *G*. *lamblia* strain could also be adapted for in vivo experimentation in infected animal models, advancing drug development and screening for *G*. *lamblia* similar to the way that Nluc expressing *C*. *parvum* has for *Cryptosporidium* [22, 23].

## Supporting information

S1 Table Pathogen Box initial screening results of *Giardia* (at 16 μM) and *Cryptosporidium* (at 10 μM). Mean percentage inhibition was calculated as the inhibition when compared to the mean values of wells grown with 1 μL DMSO in place of compound.
(XLSX)

S2 Table Validation of the EC_50_ assay results by literature. (XLSX)
